# Supplementary material for: A Combination Native Outer Membrane Vesicle (NOVM) Vaccine to Prevent Meningococcal and Gonococcal Disease
Source: Pathogens. 2025 Sep 26;14(10):979. doi: 10.3390/pathogens14100979 (PMC12566994; doi:10.3390/pathogens14100979)
Supplement: Supplementary file 1 [file pathogens-14-00979-s001.zip › pathogens-3850030-supplementary.pdf]

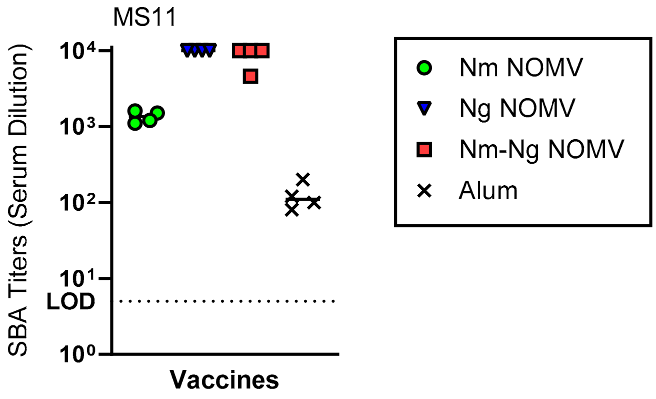

Supplementary Figure 1. SBA Titers against Ng strain MS11. SBA assays performed using IgG-IgM depleted human serum as source of complement and bacteria grown in the presence of CMP-NANA. Mouse alum sera showed SBA activity. Each symbol represents a pool from two mouse sera. Bars represent the median. Ng NOMV and Nm-Ng NOMV SBA titers were not significantly different using an unpaired T-test analysis.
